# Supplementary material for: Chronic Gut Inflammation and Dysbiosis in IBS: Unraveling Their Contribution to Atopic Dermatitis Progression
Source: Int J Mol Sci. 2024 Feb 27;25(5):2753. doi: 10.3390/ijms25052753 (PMC10931664; doi:10.3390/ijms25052753)
Supplement: Supplementary file 1 [file ijms-25-02753-s001.zip › ijms-2825613-supplementary.pdf]

Supplementary material

Chronic Gut Inflammation and Dysbiosis in IBS: Unraveling Their Contribution to Atopic Dermatitis Progression

Jae-Hwan Jang [1,†], Sun-Young Jang [2,3,†], Sora Ahn [3,4], Ju-Young Oh [3,4], Mijung Yeom [4], Seok-Jae Ko [5], Jae-Woo Park [5], Soon-Kyeong Kwon [6], Kyuseok Kim [7], In-Seon Lee [4,8], Dae-Hyun Hahm [4,9] and Hi-Joon Park [3,4,10,*]

[1] Jaseng Spine and Joint Research Institute, Jaseng Medical Foundation, Seoul, 05854, Republic of Korea; jjh2392@jaseng.org
[2] Department of Science in Korean Medicine, Graduate School, Kyung Hee University, Seoul 02447, Republic of Korea; jsy9885@khu.ac.kr (S.-Y.J.)
3 Department of Anatomy and Information Sciences, College of Korean Medicine, Kyung Hee University, Seoul 02447, Republic of Korea; ohjuyoung@khu.ac.kr; lljasmintll@gmail.com (S.A.)
[4] Acupuncture & Meridian Science Research Center, Kyung Hee University, Seoul 02447, Republic of Korea; myeom@khu.ac.kr (M.Y.); inseon.lee@khu.ac.kr (I.-S.L.); dhhahm@khu.ac.kr (D.-H.H.)
[5] Department of Gastroenterology, College of Korean Medicine, Kyung Hee University, Seoul 02447, Republic of Korea; kokokoko119@hanmail.net (S.-J.K.); pjw2907@hanmail.net (J.-W.P.)
[6] Division of Applied Life Science (BK21), Gyeongsang National University, Jinju 52828, Republic of Korea; skkwon@gnu.ac.kr
[7] Department of Ophthalmology, Otorhinolaryngology, and Dermatology of Korean Medicine, College of Korean Medicine, Kyung Hee University, Seoul 02447, Republic of Korea; kuseok@hanmail.net
[8] Department of Meridian & Acupoint, College of Korean Medicine, Kyung Hee University, Seoul 02447, Republic of Korea
[9] Department of Physiology, School of Medicine, Kyung Hee University, Seoul 02447, Republic of Korea
[10] Department of KHU-KIST Convergence Science & Technology, Kyung Hee University, 26 Kyungheedae-ro, Dongdaemun-gu, Seoul 02447, Republic of Korea
* Correspondence: acufind@khu.ac.kr
+these authors contributed equally to this work

*Corresponding author: Hi-Joon Park

Acupuncture and Meridian Science Research Center, Kyung Hee University, 26 Kyungheedaero, Dongdaemoon-gu, Seoul 02447, Republic of Korea.

E-mail: acufind@khu.ac.kr

Table S1. Detailed information on group allocation

Experiment 1: Establishment of 2,4,6-trinitrobenzenesulfonic acid (TNBS)-induced IBS mouse.

Group	Treatment
CON	Ethanol-injected group
IBS	TNBS-injected group

Experiment 2: Changes of AD symptoms in TNBS-induced IBS mouse

Group	Treatment	
	Injection into the colon	On the right cheek
CON	50% ethanol	100% ethanol
IBS	TNBS	100% ethanol
AD	50% ethanol	MC903
IBS+AD	TNBS	MC9O3

Experiment 3: Changes of AD symptoms in mice transplanted with gut microbiota from IBS mice

Group		Treatment	
		FMT	On the right cheek
CON	C-MF	transplanted with fecal microbiota from the CON mice	100% ethanol
	I-MF	transplanted with fecal microbiota from the IBS mice	
AD	C-MF	transplanted with fecal microbiota from the CON mice	MC903
	I-MF	transplanted with fecal microbiota from the IBS mice	

Experiment 4: Changes of AD symptoms in mice transplanted with gut microbiota from the IBS patient.

Group		Treatment	
		FMT	On the right cheek
CON	C-HF	transplanted from the gut microbiota of healthy control	100% ethanol
	I-HF	transplanted from the gut microbiota of the IBS patient	
AD	C-HF	transplanted from the gut microbiota of healthy control	MC903
	I-HF	transplanted from the gut microbiota of the IBS patient	

Table S2. Primer for real-time PCR

Primer	Forward	Reverse
Il-6 (Interleukin 6)	TACCACTTCACAAGTCGGAGGC	CTGCAAGTGCATCATCGTTGTTC
Il6r (Interleukin 6 receptor)	GTCATCCATGATGCCTTGCG	GTGGTCCTGGGCTCTATCCAA
Il-4 (interleukin 4)	CCCCCAGCTAGTTGTCATCC	AGGACGTTTGGCACATCCAT
Il-4r (interleukin 4 receptor)	ACGTGGTACAACCACTTCCA	TGCGGGTACTTGGTTGACTC
Mmp12 (Matrix metallopeptidase 12)	TGTATTTTGTATCTGCCTGTGGG	CTGGGGCACTGCTCTAAGAT
Il-31 (Interleukin 31)	AACAACGAAGCCTACCCTGG	TGGTTAATGCTTCCCGGTCC
Il-22 (interleukin 22)	TGCGATCTCTGATGGCTGTC	CCTCGGAACAGTTTCTCCCC
Ifn-r (interferon gamma)	GAGGTCAACAACCCACAGGT	GGGACAATCTCTTCCCCACC
Gata3 (GATA Binding Protein 3)	CCTCTGGAGGAGGAACGCTAAT	GTTTCGGGTCTGGATGCCTTCT
Il-22 (interleukin 22)	TGCGATCTCTGATGGCTGTC	CCTCGGAACAGTTTCTCCCC

Table S3. Demographics and characteristics of donor (HC and IBS)

	**HC**	**IBS**
Sex	Male	Female
Age	24	39
BMI (kh/m2)	25.5	19.1
IBS-SSS	7	313
Bristol Stool Form Scale	Normal	Type 6
IBS-D	-	65%

HC; Health control

IBS; irritable bowel syndrome
